# Potential of a laser pointer contact lens to improve the reliability of video-based eye-trackers in indoor and outdoor conditions

**DOI:** 10.16910/jemr.17.1.5

**Published:** 2024-05-16

**Authors:** François-Maël Robert, Marion Otheguy, Vincent Nourrit, Jean-Louis de Bougrenet de la Tocnaye

**Affiliations:** IMT Atlantique – Optics Dpt, France; IMT Atlantique – Optics Dpt, LaTIM, France

**Keywords:** contact lenses, eye-tracking, eye-tracker, calibration, gaze, artificial eyes

## Abstract

Many video-based eye trackers rely on detecting and tracking ocular features, a task that can be negatively affected by a number of individual or environmental factors. In this context, the aim of this study was to practically evaluate how the use of a scleral contact lens with two integrated nearinfrared lasers (denoted CLP) could improve the tracking robustness in difficult lighting conditions, particularly outdoor ones.

We assessed the ability of the CLP (on a model eye) to detect the lasers and to deduce a gaze position with an accuracy better than 1° under four lighting conditions (1 lx, 250 lx, 50 klux and alternating 1lx /250 lx) on an artificial eye. These results were compared to the ability of a commercial eye tracker (Pupil Core) to detect the pupil on human eyes with a confidence score equal to or greater than 0.9.

CLP provided good results in all conditions (tracking accuracy and detection rates). In comparison, the Pupil Core performed well in all indoor conditions (99% detection) but failed in outdoor conditions (9.85% detection).

In conclusion, the CLP presents strong potential to improve the reliability of video-based eyetrackers in outdoor conditions by providing easy trackable feature.

## Introduction

Since the second half of the twentieth century, technological progresses have allowed eye tracking to enter different fields outside the fields of neuroscience, vision or psychology (15; 6; 22; 20; 27) such as human factors (9), advertising (13), science education (Jarodzka et al., 2017) or human-computer interaction (21; 24). These same advances have also made it possible to propose new hardware and software architectures to gain in precision, speed and reliability. Among the various existing techniques: scleral search coil (31), electro-oculography, etc., video oculography is nowadays by far the dominant technique due to its non-invasive nature, relative ease of implementation and constant progresses in terms of sensors, computing power and image processing. In this approach, one or multiple camera(s) take an image of the eyes, often illuminated by infrared light sources (26). Gaze direction is then traditionally estimated using model-based methods, i.e. methods relying on a geometric eye model and analysis of pupil and/or corneal reflections.

Such methods rely on accurately detecting particular features, and consequently can be negatively affected by a number of environmental or individual factors such as ambient light, unequal eyes illumination, multiple corneal reflexes, drooping eyelids, pupil center shift, head position, eyeglasses, etc. (25; 10). Appearance based methods, i.e. methods that do not rely on any explicit segmentation stage, have been developed to tackle some of these issues and commercial applications exist (i.e. Pupil Lab’s Neon eye tracker; (18)) and benefit from advances in computing power and deep learning approaches. However, these methods can also be negatively impacted by different factors (e.g. lighting changes, scale variability; (12) and requires a large amount of data to be trained.

In this context, we have proposed to take advantage of recent results in embedded electronics to develop an electronic contact lens (19) in order to simplify the tracking. Various contact lens configurations have been presented: first using photodiodes (to have sensors whose response varies directly with eye movements (23)) then using on-board lasers that could be used to interact with position sensitive devices (29), to materialize the gaze direction (28) or simply to provide easily trackable features. These papers focused on the technical design and in each case, the lens was basically just placed on an artificial eye to validate the detection process. The aim of this study was to report functional tests with a calibrated system, and to practically assess how the use of two embedded near-infrared lasers could improve the overall sensor’s robustness to different lighting conditions when compared to a conventional wearable eye tracker.

Pupil detection, which is at the basis of the model-based approach, is actually not a trivial task (30). Various conditions such as physiological irregularities, reflection or complex illuminations can prevent a correct pupil detection and hence an accurate gaze estimation. Replacing the tracking of the pupil and possibly other elements by the tracking of two light sources should strongly simplify the problem and make it possible to track the eye even in difficult conditions such as for example outdoor illumination conditions (8; 11) that can significantly differ from classic lab conditions (e.g. large illuminance dynamic, illuminance variations associated with subject’s mobility, important infrared radiations, etc.).

The paper is organized as follow. In the Method section, we rapidly present the device, the method used to calibrate it taking advantage of the double laser configuration, the tests conditions that represent different conditions of use and that may be demanding for many eye trackers, and the protocol used. Results obtained for the different tests conditions with the contact lens eye tacker and a reference one (Pupil Lab’s core eye tracker (18)) are then presented followed by a discussion on achieved performance and future work.

## Methods

### Contact lens pointer

We refer in this article to the eye tracking system using the contact lens as the contact lens pointer (CLP). The system presented here has already been presented elsewhere but we describe it here quickly for the sake of clarity.

**Figure 1.**
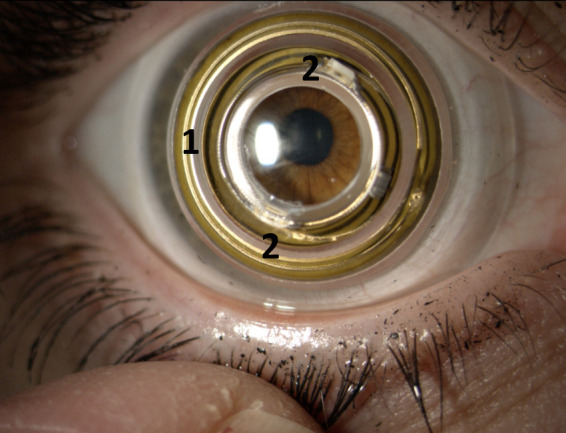
Contact lens with the secondary antenna (1) and the two VCSELs (2). The primary antenna is set in an eyewear worn by the subject. The SCL is here worn during a wearing test by the scleral lens specialist in charge of the design and manufacture of these lenses.

**Figure 2.**
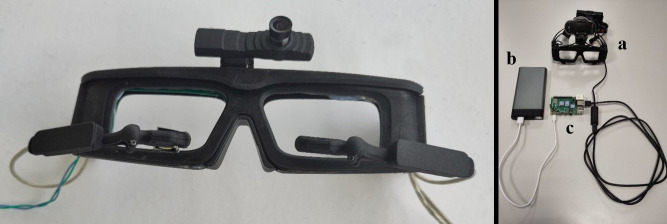
Left image: the eyewear, i.e. a 3D printed glasses frame with the primary antenna inside and supporting the Pupil Core cameras. Right image: the detection system with: a) the eyewear with an additional head-strap for improved stability, b) the battery of the Raspberry PI and c) the Raspberry Pi RP2040. This is the fully wearable configuration. When connected to a standard PC, the Raspberry and battery are not needed.

The system is made up of three parts: an instrumented scleral contact lens (Fig.1), an eyewear to power the contact lens and to take a video of the eye region (Fig.2), and a computer to process the data acquired by the camera.

The scleral contact lens is made of polymethyl methacrylate (PMMA) and has a diameter of 16.5 mm. It encapsulates a circuit comprising two vertical cavity self-emitting lasers (VCSELs) emitting at a wavelength of 850 nm, i.e. in the infrared spectrum, and a secondary antenna to power them by inductive coupling. A more precise description of the contact lens can be found in (23). Like any scleral lens, the lens is particularly stable on the eye and this point and the absence of health risks have been described in our previous papers (19; 23; 29).

The eyewear is a spectacles frame embedding a primary antenna to power the VCSELs, together with a generator and amplifier circuit to generate the signal at the right frequency and amplitude. This circuit is powered and controlled by a microcontroller card compatible with a Raspberry Pi RP2040 platform; and combined with a direct digital synthesis (DDS) chip. The DDS allows adjusting the generator to optimize the inductive transmission with the contact lens. In a conventional configuration, two cameras are mounted on the eyewear in front of the eyes to detect the VCSELs, while a third camera (world camera) records the scene seen by the user. We used here the Pupil Core architecture, from the company Pupil-Labs, for its flexibility, its open software and its performances. We added a removable IR bandpass filter to the eyes camera and covered the Pupil Core’s IR LEDs to see only the light from the contact lens’ VCSELs on the images acquired (400×400 pixels at a framerate of 120 Hz). The eyewear is 3D printed which allowed us to adjust it to position adequately the cameras.

**Figure 3.**
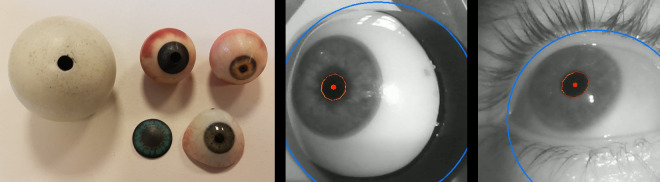
Left image: The different model eyes tested. Middle and left images: view of the eye prosthesis and a human eye when using the Pupil Core. The bright spots on the iris correspond to the Pupil Core IR source which is used to illuminate the eye.

The data from the eyes camera and world camera are transmitted to a computer through a USB connection. Because of the IR filter, the images from the eye cameras basically consist of a dark frame with only two spots corresponding to the two VCSELs. Tracking the eye therefore comes down to tracking these two posts which is simple enough to be done in real time with a Python script running on a Raspberry pi (Fig.3). The calibration step (described in the next section) allows to associate the centroids from these spots to a precise gaze direction.

### Calibration algorithm

In conventional video-based eye tracking, a calibration is required to establish a reliable correspondence between the real gaze direction and the measured features in the eye cameras images (14). In this section, we describe for the first time the algorithm used for the CLP.

Usually the participant is asked to focus their gaze on targets that appear successively at different locations in a given plane (e.g. the surface of the display monitor). The data from the eye cameras collected during this period are mapped to these specific locations using a standard configuration of the eye model (32). A successful calibration means that the collected gaze samples and the detected calibration marker, allowed to compute a 3D eye model and that the resulting mapping is correct.

The calibration procedure we use for the contact lens eye tracker establishes a relation between a given number of fixation points, represented by their coordinates in the word camera image ${(X}_{world},\;Y_{world})$, and the associated coordinates of the centroids of the two VCSELs’ spots ( $x_{left},\;y_{left})$ and ${(x}_{right},\;y_{right})$ seen by the eye camera. Mathematically, this relation can be written:

(1) $$\left(X_{world},\;Y_{world}\right)=\;f\left(x_{left},\;y_{left},\;x_{right},\;y_{right}\right)$$

Based on the literature (3; 2; 1; 17) and our own experience, we choose for *f* a second order polynomial with crossed terms, to account for the geometric dependence between VCSELs.

(2) $$X_{world}\left(x_{left},\;y_{left},\;x_{right},\;y_{right}\right)=a_{0}+a_{1}x_{left}+a_{2}y_{left}+a_{3}x_{right}+a_{4}y_{right}+a_{5}x_{left}y_{right}+a_{6}x_{right}y_{left}+a_{7}x_{left}^{2}+a_{8}y_{left}^{2}+a_{9}x_{right}^{2}+a_{10}y_{right}^{2}$$

(3) $$Y_{world}\left(x_{left},\;y_{left},\;x_{right},\;y_{right}\right)=b_{0}+b_{1}x_{left}+b_{2}y_{left}+b_{3}x_{right}+b_{4}y_{right}+b_{5}x_{left}y_{right}+b_{6}x_{right}y_{left}+b_{7}x_{left}^{2}+b_{8}y_{left}^{2}+b_{9}x_{right}^{2}+b_{10}y_{right}^{2}$$

Practical details about the calibration procedure are presented here after, after description of the test bench.

### Test Bench

In this section, we explain how and why the CLP was tested on an artificial eye when data for the Pupil Core were obtained on humans.

As previously stated, the aim of this study was to practically assess how the use of two embedded lasers could improve the overall sensor’s robustness to different lighting conditions. For this reason, tests were warried out using only one eye.

The CLP being in the process of CE certification, tests with the CLP were carried out on a model eye for ethical reasons. Ideally, all tests with the Pupil Core would have been done on the same artificial eye to ensure that measurements were made exactly in the same conditions and to avoid any uncertainties about the gaze direction. (The term "artificial eye" may suggest greater complexity than the term "model eye", but we use it here interchangeably to refer to the same element).

We tested several eye models (Fig.3a): holed table tennis ball, 3D printed scleral lens, 3D printed colored models, and finally obtained the best results with a standard ocular prosthesis set on a 3D printed eyeball (Fig. 3). Unfortunately, even though there was no noticeable difference in appearance between the eye prosthesis and the human eye in the Pupil Core images (Fig. 3), tracking performances with the Pupil Core were somehow poorer with the prothesis than with a human eye. For this reason, tests with the Pupil Core were eventually carried out on humans (using a chin rest), rather than with an artificial eye, to ensure optimum performances. In addition, to account for the fact that the quality of the measurements with the Pupil Core may depend on the user, measurements were carried out on 4 subjects, and for each test condition, only the best results were retained. (As this study involved human subjects, the approval of IMT Atlantique's ethics committee was obtained).

**Figure 4.**
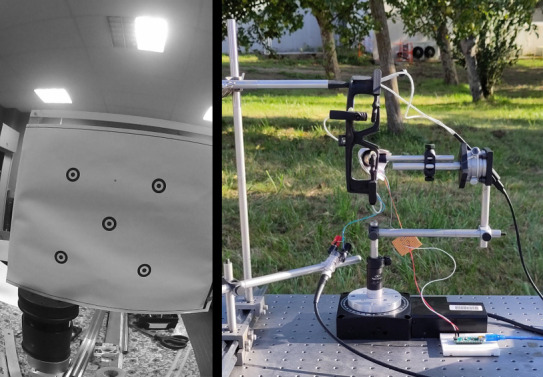
Left: view of the calibration chart by the world camera in indoor conditions. Right: the model eye on its rotating platform and driving eyewear during outdoor tests.

**Figure 5.**
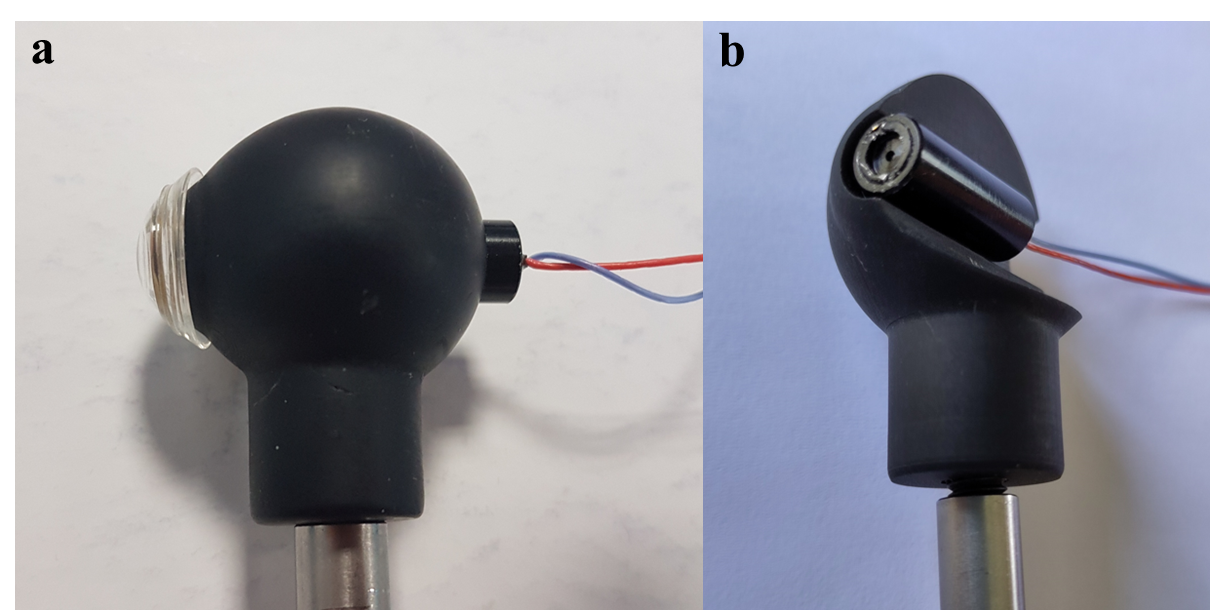
a) Model eye with an embedded laser to visualize the gaze position. b) Cross section of the model eye showing the laser.

The opto-mechanical set-up is presented in Fig. 4 next to the calibration chart as seen by the world-camera. The model eye was placed behind the eyewear (including the driving antenna and Pupil core cameras) and mounted on two rotative plates that allowed its rotation in the horizontal and vertical directions (precision of 0.5° horizontally and 0.02 degrees vertically) (Figure 4). When using the CLP, since the appearance of the iris had no importance (only the laser spots are detected) a second artificial eye was used which consisted in a 3D printed eyeball with a red laser inside it (Figure 5). This additional laser allowed visualizing directly the gaze position.

The calibration chart consists of five points placed at the extremities of a square with a 20° side and centered on (0°,0°). The calibration sequence starts with the central point and then moves from the bottom right corner to the top right one in clockwise order. When using the Pupil core, the subject sat and gazed successively at the five CP, his head immobilized by a chin rest. When calibrating the CLP, the contact lens was placed on the model eye with the embedded red laser (Fig. 5) to visualize the gaze position and the eye was rotated to gaze successively at the five CP.

### Protocol

The method used to assess the accuracy and robustness of each eye tracker when confronted with demanding lighting conditions is described below and summarized in table 1. In order to assess the potential advantage of the contact lens eye tracker in terms of robustness, four testing conditions were defined.

In the first one (C-In), lighting conditions correspond to indoor lighting (250 lux), as in a test room lit by neon lights and without windows. The second condition (C-Dark) corresponds to the case where the user is in the dark (1 lux) as could be found in some interactive environment or in some cognitive studies on the effect of darkness.

The third one (C-Alt) aims at simulating changing lighting conditions, for instance due to the user moving in a darker environment. The ambient light is alternatively turned on and out (1/250 lux) every 3s. Variations of illumination conditions could indeed impact the quality in the gaze direction’s detection in two ways: by intensity variations on the camera’s sensor (the auto-exposure algorithm may not react enough rapidly and correctly), and, in the case of a real eye, by the fact that when the pupil’s size change, the line of gaze does not necessarily intersect its center (34).

The fourth lighting condition (C-Out) corresponds to outdoor conditions on a sunny afternoon (50 klux). The eye tracker was set so that the Pupil Core cameras were not directly into the sun (Fig.4) and the user was not blinded by the sun. As in previous conditions, the photometer was held next to the front face of the eyewear. We did not measure the amount of infrared light arriving on the eye tracker because lighting measurements are traditionally given in photometric units, but it is important to note that visible light represents little more than 40% of solar radiation, and that therefore the quantity of ambient infrared was much higher than in indoors conditions.

**Table 1.**
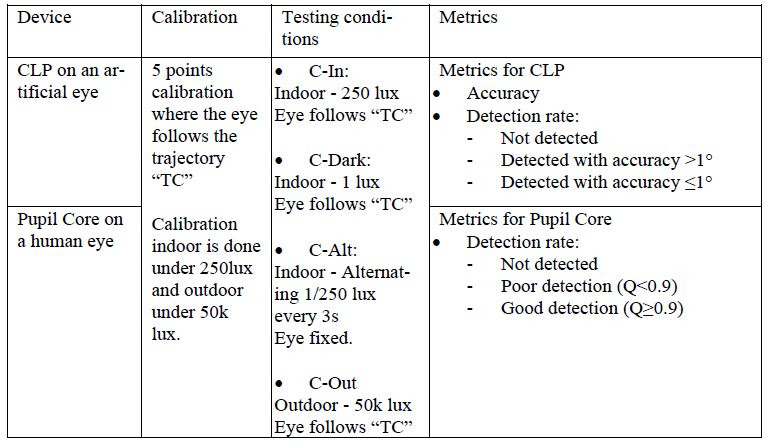
Summary of the testing protocol used in this study.

For all conditions but condition C-Alt, the eye had to follow the same trajectory as the one used during calibration. The duration of this task varies between approximately 30 seconds when using the Pupil Core and one minute when using the CLP (as the movements of the model were not fully automated). For condition C-Alt the eye stayed still and data was recorded for 20s. When using the Pupil Core, the auto-exposure function of the eye camera was activated to ensure optimum results (based on our experience). When using the CLP, such function was not implemented in our program and the exposure time was fixed prior to calibration.

Accuracy. Once our device calibrated, assessing its accuracy is straightforward. We can use the artificial eye embedding a laser pointer to point to a particular location and compare it directly to the calculated gaze position. The accuracy is then defined as the average angular offset between target and gaze position. For the Pupil Core, we simply considered the given theoretical accuracy of 0.6°.

Robustness. The surrounding illumination where the eye-tracking experience takes place (that may change rapidly in outdoor conditions or due to mobility) can decrease the performances of video-based eye-tracking systems, even if the eye is illuminated by specific light sources (IR LEDs for the Pupil Core). In the case of the Pupil Core, we first filter out blinks (using the Pupil Lab software and checking the video). Then we calculate three percentages: when the pupil is not detected (i.e. pupil diameter null), when the pupil is detected but with low confidence, and when the pupil is detected with high confidence. This confidence level is the one returned by the Pupil Core software as an assessment of the quality of the pupil detection for a given eye image, where 0 means that the pupil could not be detected and 1 when the pupil was detected with very high certainty (7). The threshold for high confidence is set arbitrarily to 0.9 which is less conservative than the 0.98 suggested by Pupil Labs (5). For the CLP, we calculate three percentages: when the laser spots are not detected, when they are detected and point to a coordinate more than 1 degree away to the true gaze position and when they are detected and point to a coordinate less than 1 degree away to the true gaze position.

## Results

**Figure 6.**
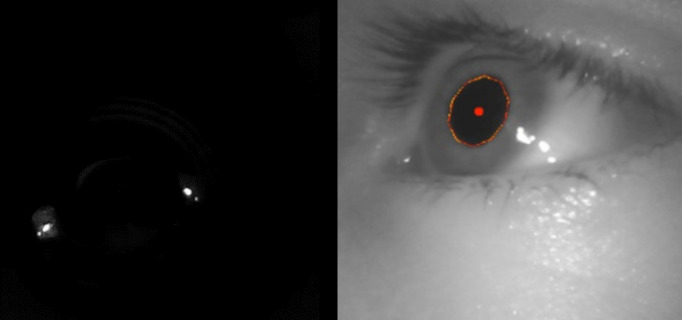
Image recorded by the Pupil Labs eye camera when using the CLP (left image) or in the classic Pupil Core configuration (right image).

**Figure 7.**
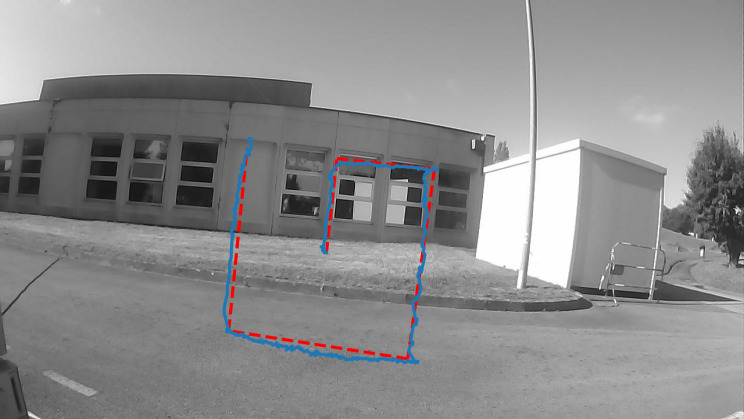
Tracking accuracy of the CLP in outdoor conditions using the calibration chart. The red line represents the trajectory (denoted “TC”) the eye has to follow. The blue line represents the gaze position calculated for the CLP.

Eye images for the CLP and Pupil Core are illustrated in Fig. 6. As expected images for the CLP are basically simple binary images with two bright spots corresponding to each VCSELs. As a result, the laser spots could be easily detected in all tested conditions (Table 2). The CLP demonstrated an accuracy equal or better than 0.27±0.27° (Table 2; Fig. 7). This result depends on the calibration model used but also on the resolution of the eye camera. In our experiment, the calibration model was not optimized and the VCSEL pair only used a small part of the CMOS sensor so a better accuracy could be easily obtained by adjusting the eye camera optics. A small percentage of gaze points were calculated with an error larger than one degree. Such points usually corresponded to the case when the image of one VCSEL spot would be saturated, leading an error on the calculation of the spot centroid.

In comparison, the Pupil Core performed well in all indoor conditions with a “Good detection” score above 99% (cf. Table 3) but failed in outdoor conditions. The poor pupil detection performances in sunlight demonstrate the difficulty to have a device that can track a passive object over a wide illumination dynamic. Good performances in condition C-In (250lux) were expected since it corresponds to a relatively classic use case for commercial eye trackers. Similar performances in the dark are not surprising since the Pupil Core uses additional IR sources to illuminate the eye. As explained in the previous section, the tracking accuracy of the pupil core was not measured. We assume it to be equal to the theoretical value when pupil detection rate is high, and worse otherwise (e.g. outdoors conditions).

Results for condition C-Alt, were in agreement with results for conditions C-In and C-Dark, i.e., the change in light conditions did not significantly impact the CLP and Pupil Core performances. For the CLP, this is not surprising since, by design the camera only receives light from the VCSELs. For the Pupil Core, this means that the auto-exposure algorithm could react rapidly and precisely enough. Temporal analysis of the data did not show that performances would decrease during lighting transitions. The fact that, in table 3, the rate of poorly detected pupils is lower in condition C-Alt than in condition C-Dark is possibly due to the variability of results associated to measurements on humans.

**Table 2.** Results with the CLP. Tracking accuracy was measured using a model eye. Pupil detection performances were measured on a model eye.

Testing conditions	Tracking accuracy (°)	No detection	Poor detection (accuracy>1°)	Good detection (accuracy≤1°)
C-In (250 lux)	0.21±0.21°	0%	2.9%	97.1%
C-Dark (1 lux)	0.15±0.24°	0%	2.7%	97.3%
C-Alt (1lx / 250 lx)	NA	0%	0%	100%
C-Out (50 klux)	0.27±0.27°	0%	0.1%	99.9%

**Table 3.** Results with the Pupil Core. Pupil detection performances were measured on a human eye.

Testing conditions	No Pupil detected (pupil diameter null)	Poor detection (Q<0.9)	Good detection (Q≥0.9)
C-In (250 lux)	0.05%	0.00 %	99.95%
C-Dark (1 lux)	0.04%	0.25%	99.71%
C-Alt (1lx / 250 lx)	0.03%	0.08%	99.89%
C-Out (50 klux)	4.25%	85.9%	9.85%

## Discussion

The first objective of this study was to report functional tests of the CLP after calibration. In terms of accuracy, our results do not show any benefit for the CLP when compared to the reported accuracy of the Pupil Core (18), although it was tested on a model eye. This is first due to the fact that they both relied on the same camera resolution. With a dedicated sensor adapted to the VCELs trajectory, higher performances should be expected. Also, in this study, we used for mapping function a generic polynomial in x and y with first order interaction. This function was chosen because it provided good performances but it may not be the most appropriate function. Other functions could be investigated based on previous work (3).

One parameter limiting the accuracy of the CLP is the high directionality of the VCSEL. As a consequence, the camera can be saturated when the VCSEL beam hits straight the sensor, leading to centroid estimation error. This directionality also limits the useful range to ±10°. This can be enough for some applications, particularly outdoors ones, i.e. when the CLP presents significant advantages over conventional eye trackers, but we also performed some tests replacing the VCSELs by a LED. The tracking range was then ±20° so large enough for any wearable application and larger than some high-end desktop eye trackers.

Another aim of the study was to assess how the use of two embedded near-infrared lasers could improve the overall sensor’s robustness to different lighting conditions when compared to a conventional wearable eye tracker.

As presented in the manuscript, the ideal method to compare both systems (CLP and Pupil Core) would have been to use a model eye to ensure that measurements were made exactly in the same conditions and to avoid any uncertainties about the gaze direction. Unfortunately, on the one hand all the various tests confirming the safety of the device had not yet been passed so the CLP had to be tested on an artificial eye. And on the other hand, we did not succeed in developing an artificial eye that would allow us to obtain with the Pupil Core results as good as with humans. Positive results with artificial eyes used to test other eyetrackers have been reported (33) but often without much detail on how the eye was made, and not for the Pupil Core, so our work may be of interest to the community.

We thus used an artificial eye for the CLP and decided to use the Pupil Core on human eyes (and retaining only the best results) because these were the most favourable conditions for the Pupil Core. This is a limitation to the study but since we only focused on the impact of the different lighting conditions on the reliability of detection of the tracking features (i.e. the VCSELs for the CLP and the pupil for the Pupil Core) we do not think that this invalidates our results, and in particular given their solidity (100% detection of CLP in conditions external vs. 9.85% for the Pupil Core). The main difference between the artificial eye and the human eye is the blink and this was accounted for in our analysis. However, a follow-up study with the CLP worn by humans is warranted to fully confirm these results.

According to our results the CLP thus presents strong potential to improve the reliability of video-based eye-trackers in outdoor conditions by providing easy trackable feature. When classic eye trackers try to find a 2D ellipse that fits the pupil, the CLP approach just relies on finding two bright spots at a known distance from one another in a binary image. Simplifying the tracking also means easier calibration, simpler and faster processing time for increased mobility and reduced latencies, and better data continuity.

 In addition to the simplicity of the stimulus on the eye camera sensor, the fact of using active tracking features helps avoid potential issues with mydriasis (4) and provide an unmissable target that stands out from any parasitic signal. This is why the CLP solution gives much better detection results in outdoor conditions than the pupil core. This increased reliability could be useful for various outdoors applications such as HMI for smart cockpits, out of home advertising or sport studies, etc.

In this study, outdoor conditions remained unchanged but in real mobility situations the wearer could move through strongly varying and complex illuminations for instance facing the sun with part of his face in the shadow. In such cases the system presented here will outperform conventional image-based eye-trackers. One solution for conventional image-based eye-trackers could be to use a very narrow spectral filter to eliminate all but the light sent by their IR light sources. However, this would naturally lead to increase the energy sent in the selected spectral band, raising potential safety issues. In addition, light from the sun is strong in all the IR part of the spectrum. The CLP could also be used in combination with existing devices. The circular antenna in the lens could then be tracked and the VCSELs turned on only in demanding situations to increase reliability.

### Ethics and Conflict of Interest

The authors declares that the contents of the article are in agreement with the ethics described in http://biblio.unibe.ch/portale/elibrary/BOP/jemr/ethics.html and that there is no conflict of interest regarding the publication of this paper.

### Acknowledgements

This research was supported in part by a grant from IMT Carnot research program and a grant from Agence Nationale de la Recherche (ANR-21-CE19-0053).

We wish to thank the company Pupil Labs for their answers to our technical questions, Bernard Abiven for manufacturing the 3D model eyes and LCS laboratoires and Vincent Ruesh for supplying the eye prostheses.
